# Sudden cessation of fluoxetine before alcohol drinking reinstatement alters microglial morphology and TLR4/inflammatory neuroadaptation in the rat brain

**DOI:** 10.1007/s00429-021-02321-9

**Published:** 2021-07-08

**Authors:** Jesús Aranda, María del Mar Fernández-Arjona, Francisco Alén, Patricia Rivera, Leticia Rubio, Inés Smith-Fernández, Francisco Javier Pavón, Antonia Serrano, Pedro J. Serrano-Castro, Fernando Rodríguez de Fonseca, Juan Suárez

**Affiliations:** 1 Instituto de Investigación Biomédica de Málaga-IBIMA, 29010 Málaga, Spain; 2 Facultad de Medicina, Universidad de Málaga, Andalucia Tech, Campus de Teatinos 32, 29071 Málaga, Spain; 3 Servicio de Farmacología Clínica, UGC Aparato Digestivo, Hospital Universitario Virgen de la Victoria, 29010 Málaga, Spain; 4 UGC Salud Mental, Hospital Regional Universitario de Málaga, 29010 Málaga, Spain; 5 Departamento de Psicobiología, Universidad Complutense de Madrid, 28223 Pozuelo de Alarcón, Spain; 6 Departamento de Anatomía Humana, Medicina Legal e Historia de la Ciencia, Universidad de Málaga, 29071 Málaga, Spain; 7 UGC Corazón, Hospital Universitario Virgen de la Victoria, 29010 Málaga, Spain; 8 UGC Neurociencias, Hospital Regional Universitario de Málaga, 29010 Málaga, Spain; 9 Red Andaluza de Investigación Clínica y Traslacional en Neurología (Neuro-RECA), Málaga, Spain; 10 Laboratorio de Investigación, Hospital Universitario Regional de Málaga, Avenida Carlos Haya 82, 29010 Málaga, Spain

**Keywords:** Alcohol, Antidepressant, Hippocampus, Inflammation, Microglia, Fractal dimension

## Abstract

Preclinical studies on the effects of abrupt cessation of selective serotonin reuptake inhibitors (SSRIs), a medication often prescribed in alcohol use disorder (AUD) patients with depression, results in alcohol consumption escalation after resuming drinking. However, a potential neuroinflammatory component on this escalation remains unexplored despite the immunomodulatory role of serotonin. Here, we utilized a rat model of 14-daily administration of the SSRI fluoxetine (10 mg/kg/day) along alcohol self-administration deprivation to study the effects of fluoxetine cessation on neuroinflammation after resuming alcohol drinking. Microglial morphology and inflammatory gene expression were analyzed in prelimbic cortex, striatum, basolateral amygdala and dorsal hippocampus. Results indicated that alcohol drinking reinstatement increased microglial IBA1 immunoreactivity and altered morphometric features of activated microglia (fractal dimension, lacunarity, density, roughness, and cell area, perimeter and circularity). Despite alcohol reinstatement, fluoxetine cessation modified microglial morphology in a brain region-specific manner, resulting in hyper-ramified (spatial complexity of branching), reactive (lower heterogeneity and circularity)-like microglia. We also found that microglial cell area correlated with changes in mRNA expression of chemokines (*Cx3cl1/fractalkine*, *Cxcl12*/*SDF1α*, *Ccl2/MCP1*), cytokines (*IL1β*, *IL6*, *IL10*) and the innate immune toll-like receptor 4 (*TLR4*) in dorsal hippocampus. Specifically, *TLR4* correlated with microglial spatial complexity assessed by fractal dimension in striatum, suggesting a role in process branching. These findings suggest that alcohol drinking reinstatement after fluoxetine treatment cessation disturbs microglial morphology and reactive phenotype associated with a TLR4/inflammatory response to alcohol in a brain region-specific manner, facts that might contribute to alcohol-induced damage through the promotion of escalation of alcohol drinking behavior.

## Introduction

Alcohol is a psychoactive substance highly consumed in the general population worldwide (Organization for Economic Co-operation and Development 2020). Individuals having uncontrolled and problematic drinking (significant distress or harm) develop a complex clinical condition called alcohol use disorder (AUD) that can cause long-lasting changes in the brain that make patients vulnerable to relapse. AUD is characterized by its association with multiple comorbidities. One-third of AUD patients have a co-occurring mental health disorder, being anxiety and/or depression the most often diagnosed (Davidson 1995; Fergusson et al. 2009; Craske and Stein 2016). Likewise, one-fourth of people with mental health problems have a co-occurring substance use disorder, most commonly, AUD. This dual diagnosis is challenging since clinical outcomes of antidepressant treatment in co-morbid dual depression is poor and there is a need for analyzing antidepressant medications in a comprehensive, integrated approach. In this regard, pharmacological interventions using selective serotonin reuptake inhibitors (SSRIs), among others (Ballesta et al. 2019, 2020), are able to reduce clinical symptoms of alcohol withdrawal including hyperlocomotion, anxiety and negative mood (Torrens et al. 2005; Uzbay 2008; Simon O’Brien et al. 2011; Bell et al. 2017). However, clinical relevance of SSRI pharmacotherapies to reduce alcohol harm is limited in AUD patients co-occurring liver and brain comorbidities such as liver steatosis, depression and/or cognitive impairment (Agabio et al. 2018; Ch’Ng and Lawrence 2018; Ray et al. 2018). Indeed, SSRI treatments (i.e., fluoxetine and sertraline) have failed to achieve efficacy in reducing alcohol drinking and alcohol relapse (Agabio et al. 2018; Kranzler et al. 1996). In addition, adherence to antidepressant medication can fail if there is relapse to alcohol use, and the harms resulting of this discontinuation are far from being understood. Our previous studies have provided evidence that fluoxetine treatment cessation during alcohol deprivation facilitates alcohol seeking escalation during drinking reinstatement (Alén et al. 2013). Recently, we have also suggested that dysregulation of glutamatergic receptor function and endocannabinoid signaling in the central amygdala likely underlies the contribution of SSRI treatment cessation in alcohol relapse (Suárez et al. 2020).

The impact of alcohol on brain functions starts with neurochemical-specific adaptations that include critical modifications in the innate neuroimmune signaling (Crews et al. 2015; Montesinos et al. 2016; Pascual et al. 2018) and changes in microglial function and phenotype involving morphology and neurochemical expression (Chastain and Sarkar 2014; Rivera et al. 2018). Microglia are highly sensitive cells that support innate immune response to alcohol through toll-like receptor 4 (TLR4) activation, a priming mechanism in ethanol-induced production of specific inflammatory cytokines (TNFα, IL1β) and chemokines (MCP1, eotaxin-1) in the brain (Pascual et al. 2018; Montesinos et al. 2015; Antón et al. 2017). Chronic overstimulation of microglia and the subsequence microglial production of cytokines including TNFα, IL1β, IL6 and TGFβ1 may contribute to neurotoxicity, neuronal cell death and brain atrophy associated with AUD (Boyadjieva and Sarkar 2010; Alfonso-Loeches and Guerri 2011; Guadagno et al. 2013). Interestingly, periods of alcohol abstinence facilitate regenerative mechanisms of neuronal recovery such as increased neurogenesis in the dentate gyrus (Morris et al. 2010). Evidence also suggests a role of TLR4 in neuropsychiatric diseases (García Bueno et al. 2016) and supports TLR4 signaling in stress-induced depression-like behaviors (Medina-Rodríguez et al. 2020; Zhang et al. 2020) and ethanol-induced long-lasting cognitive dysfunctions (Montesinos et al. 2015) that likely confer a risk of dementia in AUD patients (Crews et al. 2011). Regarding experimental models of AUD, reports coincide with clinical outcomes, providing evidence that long-term alcohol exposure reduces spatial memory and increases the production of pro-inflammatory chemokines (Ehrlich et al. 2012).

Recently, it has been hypothesized that fluoxetine might have potential neuroprotective effects through the induction of changes in both, microglial function and inflammatory responses involving TLR4/NF-kB signaling pathway (Lee et al. 2015; Liechti et al. 2015; Alboni et al. 2016; Khodanovich et al. 2018; Liu et al. 2018). Indeed, treatment with SSRIs decreases inflammatory cytokine elevations associated with major depression (Yoshimura et al. 2009; Hannestad et al. 2011; Kim et al. 2013; Chen et al. 2018), but reduces microglial response to an inflammatory stimulus with lipopolysaccharide (Tynan et al. 2012). Despite the involvement of SSRIs in the modulation of neuroinflammation and alcohol seeking behavior, whether the abrupt cessation of fluoxetine treatment participates in the innate immune response to alcohol through changes in microglia morphology and inflammatory signals is presently unknown. The present study reveals that alcohol drinking reinstatement for 3 weeks after sudden cessation of fluoxetine treatment (10 mg/kg/day for 14 days) during alcohol self-administration deprivation modifies morphometric features of reactive microglia, and this effect is tightly correlated with specific changes in mRNA expression of anti- and pro-inflammatory cytokines (*TNFα*, *IL1β*, *IL*6, *IL4*, *IL10*, *TGFβ*, *BDNF*), chemokines (*Cx3cl1*, *Cxcl12*, *Ccl2*) and *TLR4* in a rat brain region-specific manner (prelimbic cortex, striatum, basolateral amygdala and dorsal hippocampus). Animals self-administering saccharine, receiving the same pattern of fluoxetine treatment, were used as control group.

## Materials and methods

### Ethics statements

The protocols for animal care and use were approved by the Ethics and Research Committee at the University of Malaga (CEUMA registry no. 84–2015-A). All experimental procedures were performed in strict accordance with the Directive 2010/63/EU of the European Parliament and the EU Council (22 September 2010) and the Spanish regulation (RD 53/2013) on the protection of animals used for scientific purposes. We carried out the experiments in strict terms to minimize animal suffering and to reduce the number of animals used. Animal studies comply with the ARRIVE guidelines.

### Animals

Thirty-eight male Wistar rats (Crl:WI, Charles Rivers, Barcelona, Spain), weighting 250 g and aging 10–12 weeks at the beginning of the experiments, were housed and allowed to acclimatize in standard conditions at 23 ± 1 °C room temperature, 40 ± 5% relative humidity and a 12-h light–dark cycle with dawn/dusk effect. Tap water and standard rodent chow (Prolab RMH 2500, 2.9 kcal/g) were available ad libitum. The animals had 2 weeks for the adjustment of the environmental conditions, before the implementation of the self-administration training protocol.

### Drugs

Alcohol solution (10% alcohol w/v solution) was prepared daily from 99% ethanol. Solution of fluoxetine HCl (Prozac®, Eli Lilly, Alcobendas, Spain) was also prepared daily by dissolving in 0.9% saline and were injected intraperitoneally (i.p.) at a concentration of 10 mg/kg in a volume of 2 mL/kg body weight as previously described (Alén et al. 2013; Suárez et al. 2020).

### Operant alcohol self-administration

**Fig. 1:**
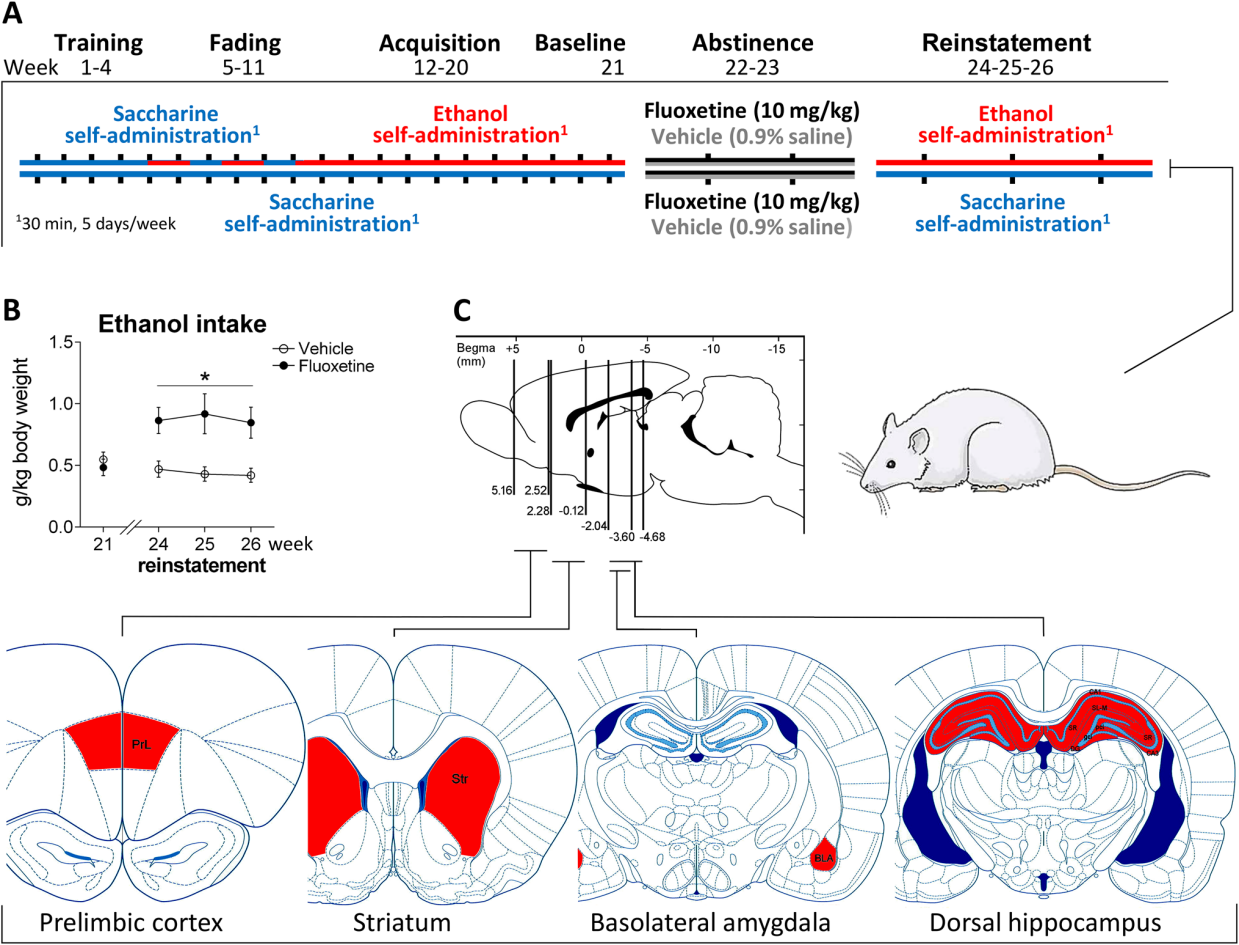
Schematic representation of the experiment. (**A**) Timeline of a paradigm of a rat model of ethanol self-administration showing stable ethanol drinking after 21 weeks of acquisition following abstinence and fluoxetine treatment (10 mg/kg/day) for 14 days, and ethanol drinking reinstatement for 3 weeks. (**B**) Weekly average of ethanol intake (g/kg) were represented during ethanol self-administration baseline (week 21) and reinstatement (weeks 24–26). Tukey’s test: *p < 0.05 *vs*. vehicle. (**C**) Schematic representation of the brain regions analyzed

A total of 26 animals were trained according to a paradigm of operant ethanol self-administration as previously described (Ballesta et al. 2019; Suárez et al. 2020). We used an alcohol relapse model based on the alcohol deprivation effect, which consisted of excellent face and predictive validity in relation to alcohol consumption supported by previous studies (Ballesta et al. 2019; Alén et al. 2013; Suárez et al. 2020; Vengeliene et al. 2014). Briefly, after lever-press training habituation by using a 0.2% saccharine facing procedure (Alén et al. 2013) in operant cages set with liquid dispenser (LE850W, Panlab, Barcelona, Spain), all animals were then introduced in alcohol operant sessions consisted of 30 min/day over a 5 day/week (Monday to Friday) following a proportional sequence of saccharine/ethanol (0.16/2 for 3 sessions; 0.12/4 for 3 sessions; 0.08/6 for 4 sessions; 0.04/8 for 4 sessions; 0.02/10 for 4 sessions; and finally 10% ethanol for the remaining sessions) on a fixed-ratio 1 schedule until steady levels of alcohol self-administration were achieved (baseline, 30 presses with less than 15% variation in 2 consecutive days). Pressing the active lever resulted in the delivery of 0.1 mL of the ethanol solution (time-out of 2.5 s). After that alcohol self-administration was withdrawn (abstinence) and the 26 animals were randomly assigned to be daily treated with fluoxetine (10 mg/kg) or vehicle (saline) for 14 days (*n* = 13/group). Then, 24 h after treatment was ceased, alcohol self-administration sessions of 30 min/day were re-introduced (reinstatement) for 3 consecutive weeks (Fig. 1A). Twelve additional rats were trained for saccharine self-administration following the same schedule and operant sessions as those followed by the ethanol-exposed rats. In the same way, rats were randomly treated with fluoxetine (10 mg/kg) or vehicle (saline) for 14 days (*n* = 6/group). Finally, saccharine self-administration sessions were re-introduced for 3 weeks.

### Sample collection

One hour after the last alcohol/saccharine self-administration session, all animals were anaesthetized with sodium pentobarbital (50 mg/kg, i.p.). Some animals (*n* = 6/group) were perfused transcardially with 4% formaldehyde in 0.1 M phosphate buffer (PB). Whole brains were extracted and processed for − 80 °C cryopreservation with 30% sucrose in PB. The brains were then cut into 30 μm-thick coronal sections by using a freezing microtome (Leica SM2000R). Sections were stored at 4 °C in PB with 0.002% (*w/v*) sodium azide until they were used for immunohistochemical analysis. Remaining animals (*n* = 7/group) were killed by decapitation, brains were collected, and the prelimbic cortex (PrL, also called medial prefrontal cortex), striatum (Str), basolateral amygdala (BLA) and dorsal hippocampus were dissected and stored at − 80 °C until they were used for mRNA expression analysis by RT-qPCR technique.

### Immunohistochemistry

Free-floating coronal sections were selected from 5.16 to 2.52 mm, 2.28 to − 0.12 mm, − 2.04 to − 3.60 mm and − 2.04 to − 4.68 mm of Bregma levels (Paxinos and Watson 2007), corresponding to PrL, Str, BLA and dorsal hippocampus, respectively (Fig. 1C). A minimum of eight sections per brain region were selected from one of the six parallel series obtained from each rat brain of the four experimental groups (n = 6/group). Sections were immunostained simultaneously using a four-well plate (aprox. 48 sections/well). Sections were incubated overnight at 4 °C in the following diluted primary antibody: polyclonal rabbit anti-IBA-1 (1:1000, cat. no. 019–19,741, Wako, Neuss, Germany. RRID: AB_839504). After antibody incubation, the sections were washed and incubated in biotinylated donkey anti-rabbit IgG (1:500, cat. no. RPN1004, GE Healthcare, Barcelona, Spain. RRID: AB_1062582) at room temperature for 2 h. Then, the sections were incubated in ExtrAvidin®-Peroxidase (1:2000, cat. no. E2886, Sigma-Aldrich-Merck, Darmstadt, Germany. RRID: AB_2620165) in darkness at room temperature for 1 h. Finally, immunolabeling was revealed with 0.05% diaminobenzidine (DAB, Sigma-Aldrich), 0.05% nickel ammonium sulfate (Ni) and 0.03% H_2_O_2_ in PB-saline for at least 10 min.

### Stereological cell quantification

Representative counting frames were obtained using a standard optical microscope equipped with the 40 × objective (Nikon Instruments Europe B.V., Amstelveen, The Netherlands) and coupled to the NIS-Elements Imaging Software 3.00 (Nikon). The brain regions of interest for this study were the following: PrL, Str, BLA, and the hippocampal areas CA1 and CA3 and the dentate gyrus. A minimum of eight coronal sections per brain region were analyzed, which resulted in one of every six equidistant sections (one representative section for each 180 µm) according to the rostro-caudal extent. Estimations of the number of IBA-1-immunoreactive ( +) cells per area (mm^2^) in both hemispheres were manually counted according to stereological methods and calculated based on the following formula (Rivera et al., 2018):

$$Na~ = ~\Sigma \left( {Q - } \right)/\Sigma \left( {astr} \right),$$

where ΣQ − is the total number of positive ( +) cells counted per animal, and *a*_*str*_ is the area (mm^2^) of the structure analyzed. Cell number quantification was expressed as the average number of IBA-1 + cells per area (mm^2^) for each experimental group. For densitometric analysis, quantification of IBA1 immunoreactivity is determined using ImageJ software (NIH, USA) and expressed as arbitrary units of average intensity of the image field that corresponds to representative areas of the brain regions.

### Morphometric analysis

Image acquisition was carried out with the aim of morphometric analysis of microglial cells. For this purpose, digital color images of PrL, Str, BLA and CA1 sections immunolabeled with IBA-1 antibody and DAB-Ni were obtained using an Olympus VS120 microscope. The UPLSAPO 60xO oil immersion objective was used to capture high-resolution images (pixel size side = 115 nm) of the selected areas. A multi-plane virtual-Z mode allowed to capture 20 images (1 µm thick) in 20 µm depth of the tissue section, which were later combined to obtain a single high-quality image including detailed magnification of ramified processes of the cells. Each obtained image was a TIFF file of 96 ppi and contained at least 30 cells. These images were cropped to delimit single cells according to the following criteria: (i-a) random selection in prefrontal cortex areas corresponding to the prelimbic cortex (PrL); (i-b) random selection starting from the area nearest to the lateral ventricle (LV) towards the brain parenchyma up to a depth of about 100 µm into the dorsal part of the striatum (Str); (i-c) random selection in the basolateral amygdala (BLA); and (i-d) random selection throughout the striati radiatum (SR) and lacunosum-moleculare (SL-M) of the hippocampal CA1 region; (ii) no overlapping with neighboring cells; and (iii) complete nucleus and branches (at least apparently). Selection was blinded to alcohol and treatment. Fifty cells randomly selected from six animals per experimental group and brain region were analyzed. A total of 800 images was evaluated. Each single cell image was processed in a systematic way using FIJI free software, an application from the ImageJ software. After the image processing of each single cell, we obtained a “filled image” and its counterpart “outlined shape” as previously described (Rivera et al. 2018; Fernández-Arjona et al. 2017). These filled and outlined cell images were subsequently used for morphometric analysis. Morphological features of microglial cells were quantified with the free software FracLac for ImageJ ((Karperien et al. 2013); FracLac for ImageJ; available at the ImageJ website, NIH). The following 15 parameters were measured as previously described (Fernández-Arjona et al. 2017): (1) *fractal dimension* (D); (2) *lacunarity* (Ʌ); (3) *cell area* (CA, μm^2^); (4) *cell perimeter* (CP, μm); (5) *cell circularity*; $[CC = (4\pi \cdot CA)/(CP)2]$ (6) *convex hull area* (CHA, μm^2^); (7) *density* (ρ, CA/CHA); (8) *convex hull perimeter* (CHP, μm); (9) *roughness* (CP/CHP); (10) *convex hull circularity;* $[CHC = (4\pi \cdot CHA)/(CHP)2]$ (11) *convex hull span ratio* (CHSR, M/m); (12) *diameter of the bounding circle* (BCD, μm); (13) *maximum span across the convex hull* (MSACH, μm); (14) *the ratio maximum/minimum convex hull radii* (RCHR, R/r); and (15) *the mean radius* (MR, μm).

### RNA isolation and RT-qPCR analysis

RT-qPCR (Taqman, Applied Biosystem, Carlbad, CA, USA) was performed in a CFX96TM Real-Time PCR Detection System (Bio-Rad, Hercules, CA, USA), using specific sets of primer probes (Table 1) and the FAM dye label format for the Taqman® Gene Expression Assays (ThermoFisher), as described previously (Rivera et al., 2018). Briefly, Prl, Str, BLA and dorsal hippocampal samples were homogenized on ice and RNA was extracted following Trizol® method according to the manufacture’s instruction (Gibco BRL Life Technologies, Baltimore, MD, USA). RNA was isolated, and reverse transcript reaction (RT, 1 µg of RNA) and quantitative real-time reverse transcription polymerase chain reaction (qPCR) were performed. Melting curve analysis was obtained to ensure that only a single product was amplified. Ct values from mRNA expression of inflammatory factors were normalized in relation to those Ct values from *Gapdh* mRNA expression.

**Table 1:** Primer references for TaqMan® Gene Expression Assays (ThermoFisher)

Gene ID	GenBank accession numbers	Assay ID	Amplicon length
*CX3CL1 (Fractalkine)*	NM_134455.1	Rn00593186_m1	74
*CXCL12 (SDF1)*	NM_001033882.1	Rn00573260_m1	60
*CCL2 (MCP-1)*	NM_031530.1	Rn00580555_m1	95
*TNFα*	NM_012675.3	Rn01525859_g1	92
*IL1β*	NM_031512.2	Rn00580432_m1	74
*IL-6*	NM_012589.2	Rn01410330_m1	121
*IL-4*	NM_201270.1	Rn01456866_m1	128
*IL-10*	NM_012854.2	Rn01483988_g1	105
*TGFB1*	NM_021578.2	Rn00572010_m1	65
*BDNF*	NM_001270630.1	Rn02531967_s1	142
*TLR4*	NM_019178.1	Rn00569848_m1	127
*Gadph*	NM_017008.4	Rn01775763_g1	174

*BDNF*: Brain-derived Neurotrophic factor; *CCL2/MCP1*: Monocyte chemoattractant protein-1; *CX3CL1*: Fractalkine; *CXCL12/SDF1*: Stromal cell-derived factor 1; *Gapdh*: Glyceraldehyde-3-phosphate dehydrogenase; *IL1β*: Interleukin 1 beta; *IL-4*: Interleukin 4; *IL-6*: Interleukin 6; *IL-10*: Interleukin 10; *TGFB1*: Transforming growth factor beta 1; *TLR4:* Toll-like receptor 4; *TNFα*: Tumor necrosis factor

### Statistical analysis

Comparisons of data were carried out by using GraphPad Prism 6.0 (GraphPad Software, San Diego, CA, USA). The data on IBA-1 immunoreactivity, IBA-1 + cell number per area (mm^2^), morphometric parameters of microglia, and relative mRNA expression of inflammatory factors were represented as means ± Standard Error of the Mean (SEM). The n indicates the number of animals (*n* = 6–7) or cells recorded (*n* = 50) per experimental group. Kolmogorov–Smirnov normality test was used to verify Gaussian distribution. Statistical significance for IBA-1 immunohistochemistry and microglial morphology were determined by repeated/two-way ANOVA with time (weeks), drinking (saccharine *vs*. ethanol) and treatment (vehicle *vs*. fluoxetine) as main factors, followed by Tukey’s post hoc tests for multiple comparison, where appropriate. Statistical significance for mRNA expression was determined by unpaired Student’s *t*-test. To analyze whether fluoxetine treatment cessation induced changes in the association between parameters, correlation model analysis was performed and *Rho* values (goodness-of-fit) calculated. A *P* value less than 0.05 indicates statistical significance.

## Results

### Fluoxetine treatment cessation increases ethanol self-administration during reinstatement

As expected, a significant increase in weekly ethanol self-administration during the re-exposure period (reinstatement) was detected in the rats that were previously treated with fluoxetine during the ethanol deprivation period (Fig. 1B). Repeated measures ANOVA indicated an overall treatment effect on ethanol self-administration for 3 weeks (*F*_1,72_ = 26.66, *p* < 0.0001), with fluoxetine-treated rats having higher consumption of ethanol during reinstatement (post hoc comparisons: *p < 0.05 *vs*. ethanol-exposed rats treated with vehicle). Interaction between time and treatment was found when baseline period was introduced in the statistical analysis (*F*_3,96_ = 3.56, *p* < 0.02), suggesting that fluoxetine treatment cessation modified ethanol self-administration during reinstatement compared to ethanol self-administration baseline.

### Ethanol drinking reinstatement increases IBA-1 immunoreactivity in the brain

To analyze the effects of the abrupt cessation of fluoxetine treatment during ethanol abstinence and ethanol drinking reinstatement on the presence of microglia in the rat brain, we quantified the IBA-1 + cell number and IBA-1 immunoreactivity (intensity) in the PrL, Str, BLA, and the hippocampal areas CA1 and CA3, and the dentate gyrus.

**Fig. 2:**
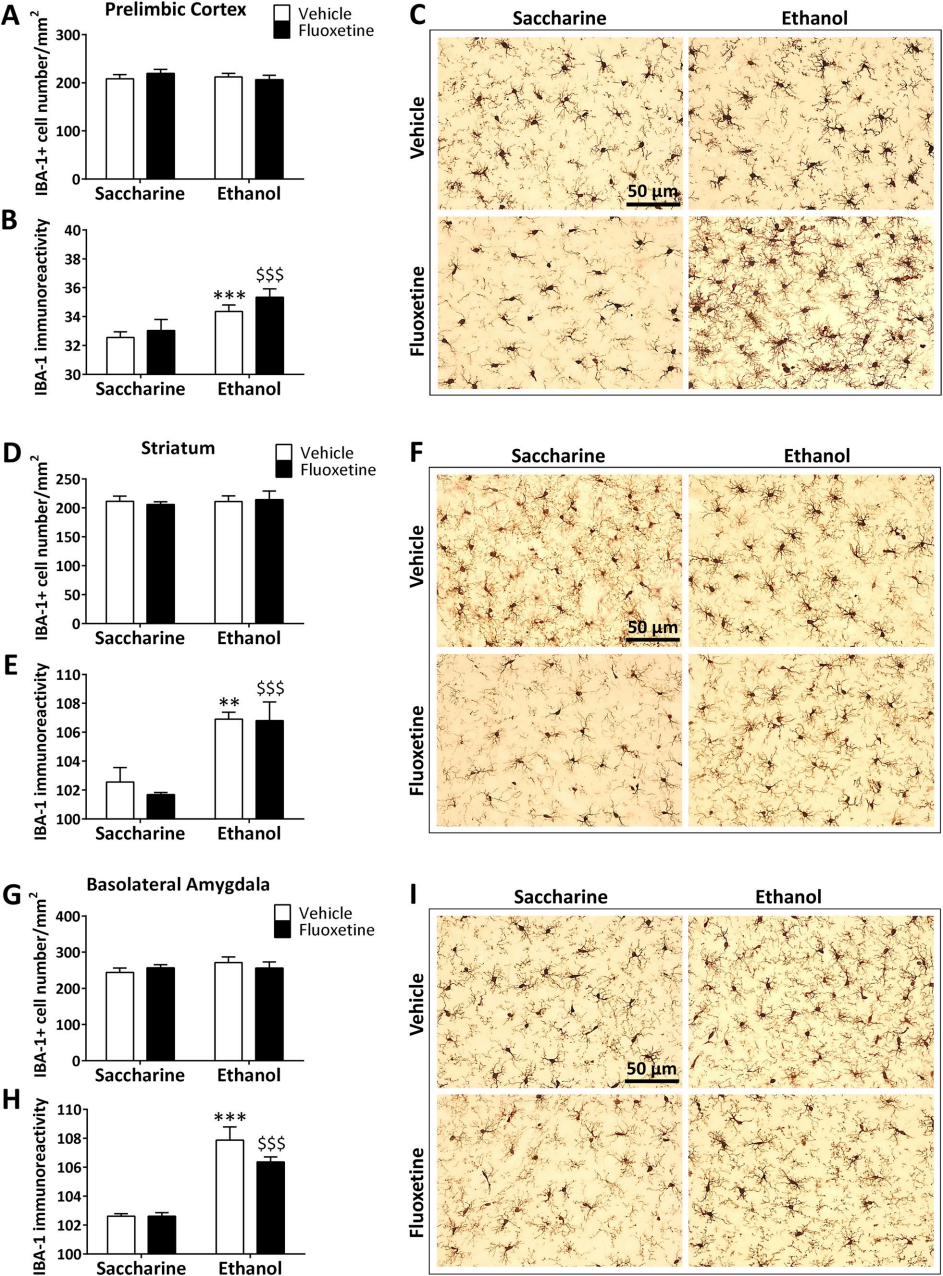
Effects of fluoxetine treatment cessation and ethanol drinking reinstatement on the number of IBA-1 + microglial cells and IBA-1 immunoreactivity in the prelimbic cortex (**A**, **B**), striatum (**D**, **E**) and basolateral amygdala (**G**, **H**). The histograms represent the mean + SEM of cells per area (mm^2^) and arbitrary units of immunoreactivity (*n* = 6 rats per experimental group). Tukey’s test: **/***p < 0.01/0.001 *vs*. saccharine-vehicle group; ^$$$^*p* < 0.001 *vs*. saccharine-fluoxetine group. Representative microphotographs showing magnification views of the immunostaining in the prelimbic cortex (**C**), striatum (**F**) and basolateral amygdala (**I**). Scale bars are included in representative images

Alcohol, but not saccharine drinking, induced significant overall effects on the IBA-1 immunoreactivity, but not IBA-1 + cell number, in the PrL (*F*_1,20_ = 12.13, *p* = 0.0027), Str (*F*_1,20_ = 29.88, *p* < 0.0001) and BLA (*F*_1,20_ = 46.08, *p* < 0.0001), showing a higher intensity in both ethanol-exposed rats (post hoc comparisons: **^/^***p < 0.01/0.001 *vs*. saccharine-exposed rats) and ethanol-exposed rats treated with fluoxetine (post hoc comparisons: ^$$$^*p* < 0.001 *vs*. saccharine-exposed rats treated with fluoxetine, Fig. 2A–I). Treatment overall effect and interaction between drinking and treatment were not detected in the three brain regions.

**Fig. 3:**
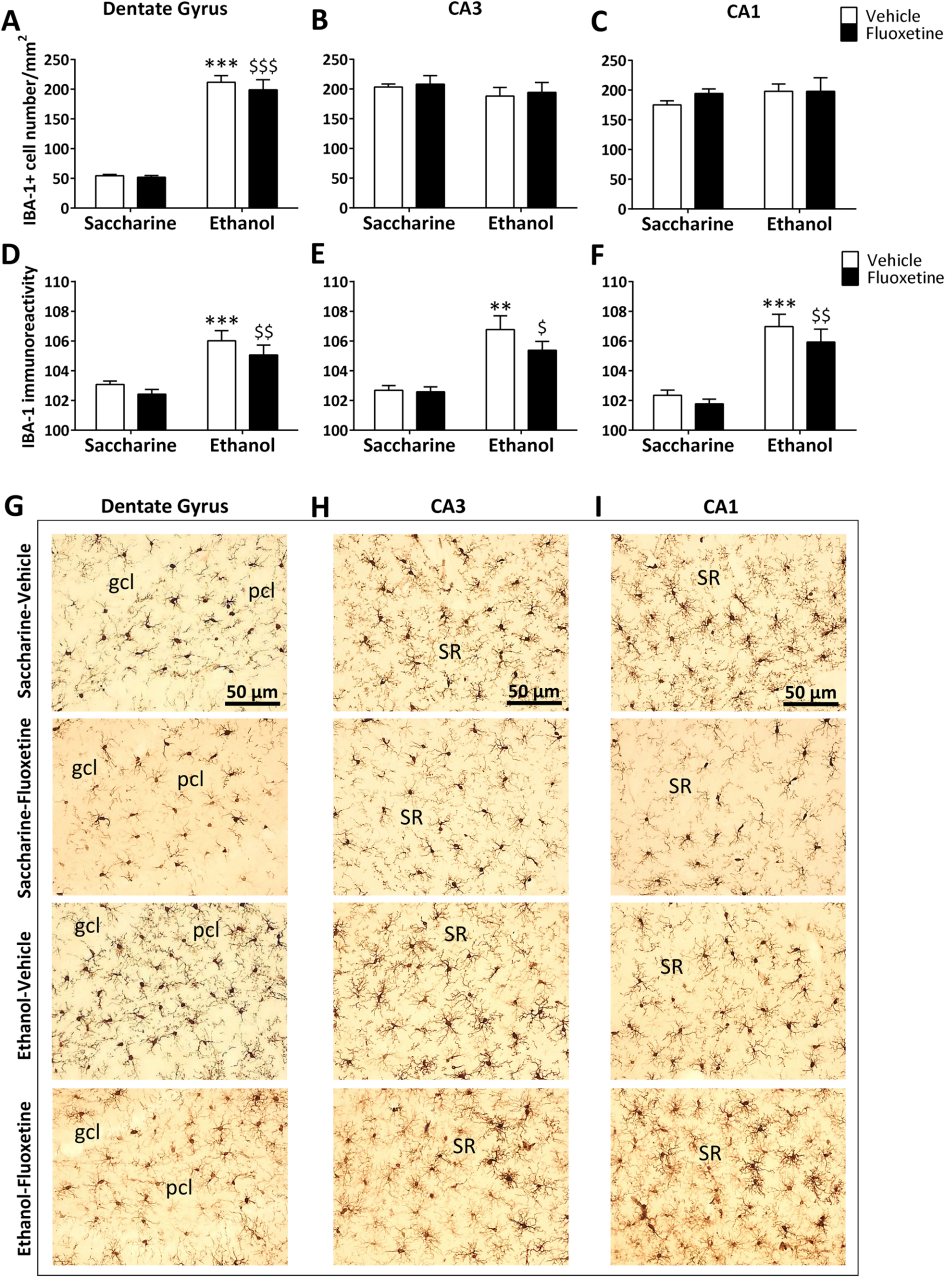
Effects of fluoxetine treatment cessation and ethanol drinking reinstatement on the number of IBA-1 + microglial cells and IBA-1 immunoreactivity in the dentate gyrus (**A**, **D**), and the hippocampal CA3 (**B**, **E**) and CA1 (**C**, **F**). The histograms represent the mean + SEM of cells per area (mm^2^) and arbitrary units of immunoreactivity (*n* = 6 rats per experimental group). Tukey’s test: **/***p < 0.01/0.001 *vs*. saccharine-vehicle group; ^$/$$/$$$^*p* < 0.05/0.01/0.001 *vs*. saccharine-fluoxetine group. Representative microphotographs showing magnification views of the immunostaining in the dentate gyrus (**G**), and the hippocampal CA3 (**H**) and CA1 (**I**). Scale bars are included in representative images

Drinking induced a significant overall effect on IBA-1 + cell number in the dorsal hippocampus (*F*_1,20_ = 15.03, *p* = 0.001), showing a specific increase in the dentate gyrus (overall effect: *F*_1,20_ = 154.4, *p* < 0.0001), but not CA3 and CA1 areas, of both ethanol-exposed rats (post hoc comparisons: ***p < 0.001 *vs*. saccharine-exposed rats) and ethanol-exposed rats treated with fluoxetine (post hoc comparisons: ^$$$^*p* < 0.001 *vs*. saccharine-exposed rats treated with fluoxetine, Fig. 3A–C). Drinking also resulted in an overall effect on IBA-1 immunoreactivity in the dorsal hippocampus (*F*_1,20_ = 31.36, *p* < 0.0001), including dentate gyrus (*F*_1,20_ = 21.41, *p* < 0.001), CA3 (*F*_1,20_ = 22.15, *p* < 0.001) and CA1 (*F*_1,20_ = 32.67, *p* < 0.0001). As a consequence, ethanol increased IBA-1 immunoreactivity in these hippocampal regions of both ethanol-exposed rats (post hoc comparisons: **^/^***p < 0.01/0.001 *vs*. saccharine-exposed rats) and ethanol-exposed rats treated with fluoxetine (post hoc comparisons: ^$/$$^*p* < 0.05/0.01 *vs*. saccharine-exposed rats treated with fluoxetine, Fig. 3D–F). Treatment overall effect and interaction between drinking and treatment were not detected in dorsal hippocampus. Representative images of the immunohistochemical expression of IBA-1 and apparent density of IBA-1 + cells are shown in Fig. 3G–I.

### Fluoxetine treatment cessation before ethanol drinking reinstatement modifies microglial morphology in a brain region-specific manner

We assessed whether increases in IBA-1 immunoreactivity induced by ethanol are associated with changes in morphometric parameters of microglial cells expressing IBA-1 in the PrL, Str, BLA and the hippocampal CA1 region. We also evaluated whether fluoxetine treatment cessation modifies the putative ethanol effect on microglial morphology.

#### Analysis of interaction and main overall effects of drinking and treatment

Significant overall effects of drinking (saccharine *vs*. ethanol) on fractal dimension (spatial complexity), lacunarity (heterogeneity), roughness (process surface and branching), and cell area, perimeter and circularity were detected in the microglia of the PrL (Table 2). We also observed significant overall effects of drinking on most morphometric parameters, excepting lacunarity, convex hull span ratio (CHSR) and the maximum/minimum convex hull radii (RCHR), in the microglia of the Str. Significant effects of drinking on lacunarity and density (process shortening/thickening) were only observed in the microglia of the BLA. Finally, significant overall effects of drinking on most morphometric parameters, excepting lacunarity, density, cell circularity, convex hull circularity (CHC), bounding circle diameter (BCD) and maximum span across the convex hull (MSACH), were observed in the microglia of the hippocampal CA1 region (Table 2).

**Table 2:** Interaction and effects of drinking (saccharine *vs*. ethanol) and treatment (vehicle *vs*. fluoxetine) on morphometric parameters of microglial cells expressing IBA-1

Morphometric parameters	Two-way ANOVA	Prelimbic cortex (PrL)	Striatum (Str)	Basolateral amygdala (BLA)	Hippocampal CA1 region
Fractal dimension (D)	Interaction Drinking Treatment	*ns*^*a*^ *F*_1,196_ = 4.88, *P* < .03 *ns*	*ns* *F*_1,196_ = 11.2, *P* < .001 *F*_1,196_ = 6.34, *P* < .02	*ns* *ns* *ns*	*F*_1,196_ = 5.93, *P* < .02 *F*_1,196_ = 6.10, *P* < .02 *F*_1,196_ = 4.65, *P* < .03
Lacunarity (Ʌ)	Interaction Drinking Treatment	*ns* *F*_1,196_ = 12.1, *P* < .001 *ns*	*ns* *ns* *ns*	*ns* *F*_1,196_ = 3.75, *P* = .05 *ns*	*F*_1,196_ = 13.9, *P* < .001 *ns* *ns*
Cell area (CA)	Interaction Drinking Treatment	*ns* *F*_1,196_ = 7.27, *P* < .01 *ns*	*ns* *F*_1,196_ = 18.4, *P* < .000 *ns*	*ns* *ns* *ns*	*ns* *F*_1,196_ = 25.4, *P* < .000 *ns*
Cell perimeter (CP)	Interaction Drinking Treatment	*ns* *F*_1,196_ = 7.67, *P* < .01 *ns*	*ns* *F*_1,196_ = 10.8, *P* < .001 *F*_1,196_ = 4.19, *P* < .04	*F*_1,196_ = 3.13, *P* = .07 *ns* *ns*	*ns* *F*_1,196_ = 21.2, *P* < .000 *ns*
Cell circularity (CC)	Interaction Drinking Treatment	*ns* *F*_1,196_ = 9.42, *P* < .01 *ns*	*F*_1,196_ = 4.25, *P* < .04 *F*_1,196_ = 11.1, *P* < .001 *F*_1,196_ = 4.69, *P* < .03	*F*_1,196_ = 4.49, *P* < .03 *ns* *ns*	*ns* *ns* *F*_1,196_ = 11.2, *P* < .001
Convex hull area (CHA)	Interaction Drinking Treatment	*ns* *ns* *ns*	*ns* *F*_1,196_ = 5.00, *P* < .03 *F*_1,196_ = 5.03, *P* < .03	*F*_1,196_ = 3.69, *P* = .05 *ns* *ns*	*ns* *F*_1,196_ = 11.6, *P* < .001 *F*_1,196_ = 6.54, *P* < .02
Density (ρ)	Interaction Drinking Treatment	*ns* *ns* *ns*	*ns* *F*_1,196_ = 8.77, *P* < .003 *ns*	*ns* *F*_1,196_ = 8.52, *P* < .004 *ns*	*F*_1,196_ = 11.4, *P* < .01 *ns* *F*_1,196_ = 5.38, *P* < .03
Convex hull perimeter (CHP)	Interaction Drinking Treatment	*ns* *ns* *ns*	*ns* *F*_1,196_ = 5.84, *P* < .02 *F*_1,196_ = 4.20, *P* < .04	*F*_1,196_ = 3.52, *P* = .06 *ns* *ns*	*ns* *F*_1,196_ = 6.96, *P* < .01 *F*_1,196_ = 6.76, *P* < .02
Roughness (R)	Interaction Drinking Treatment	*F*_1,196_ = 4.10, *P* < .05 *F*_1,196_ = 8.15, *P* < .01 *ns*	*ns* *F*_1,196_ = 10.6, *P* < .002 *F*_1,196_ = 4.22, *P* < .04	*ns* *ns* *ns*	*ns* *F*_1,196_ = 9.41, *P* < .003 *ns*
Convex hull circularity (CHC)	Interaction Drinking Treatment	*ns* *ns* *ns*	*ns* *F*_1,196_ = 4.05, *P* < .05 *ns*	*ns* *ns* *ns*	*ns* *ns* *F*_1,196_ = 34.4, *P* < .000
Convex hull span ratio (CHSR)	Interaction Drinking Treatment	*ns* *ns* *ns*	*ns* *ns* *ns*	*ns* *ns* *ns*	*ns* *F*_1,196_ = 32.4, *P* < .000 *ns*
Bounding circle diameter (BCD)	Interaction Drinking Treatment	*ns* *ns* *F*_1,196_ = 6.10, *P* < .05	*ns* *F*_1,196_ = 5.10, *P* < .03 *ns*	*ns* *ns* *ns*	*ns* *ns* *F*_1,196_ = 6.96, *P* < .01
Maximum span across the convex hull (MSACH)	Interaction Drinking Treatment	*ns* *ns* *F*_1,196_ = 6.16, *P* < .05	*ns* *F*_1,196_ = 4.98, *P* < .03 *ns*	*ns* *ns* *ns*	*ns* *ns* *F*_1,196_ = 6.74, *P* < .02
The ratio maximum/ minimum convex hull radii (RCHR)	Interaction Drinking Treatment	*ns* *ns* *ns*	*ns* *ns* *ns*	*ns* *ns* *ns*	*ns* *F*_1,196_ = 26.3, *P* < .000 *ns*
The mean radius (MR)	Interaction Drinking Treatment	*ns* *ns* *F*_1,196_ = 4.36, *P* = .05	*ns* *F*_1,196_ = 5.63, *P* < .02 *ns*	*ns* *ns* *ns*	*ns* *F*_1,196_ = 5.32, *P* < .03 *F*_1,196_ = 7.59, *P* < .007

ns, not significant

Significant overall effects of treatment (vehicle *vs*. fluoxetine) on BCD, MSACH and the mean radius (MR) were detected in the microglia of the PrL, and no treatment effect on the morphometric parameters in the microglia of the BLA was found (Table 2). Prominently, we found significant overall effects of treatment on fractal dimension, roughness, cell perimeter and circularity, and convex hull area and perimeter in the microglia of the Str. Significant overall treatment effects on fractal dimension, cell circularity, convex hull area, perimeter and circularity, density, BCD, MSACH and MR were also found in the microglia of the hippocampal CA1 region (Table 2).

Interaction between factors (drinking *vs*. treatment) was only observed in roughness in the microglia of the PrL, suggesting a specific increase in process surface and branching in ethanol-exposed rats that were previously treated with fluoxetine. Regarding the striatum, interaction was mainly detected in microglial cell circularity (Table 2), suggesting that fluoxetine decreases the proportion between microglial cell area and perimeter in a drinking-dependent manner. In the microglia of CA1 region, interaction was found in fractal dimension, lacunarity and density, with fluoxetine decreasing spatial complexity and increasing heterogeneity and process length/thinning in a drinking-dependent manner. Interaction was not observed when morphometric parameters of microglia were analyzed in BLA (Table 2).

#### Simple effect analysis of fractal dimension, lacunarity, and cell area, perimeter and circularity

**Fig. 4:**
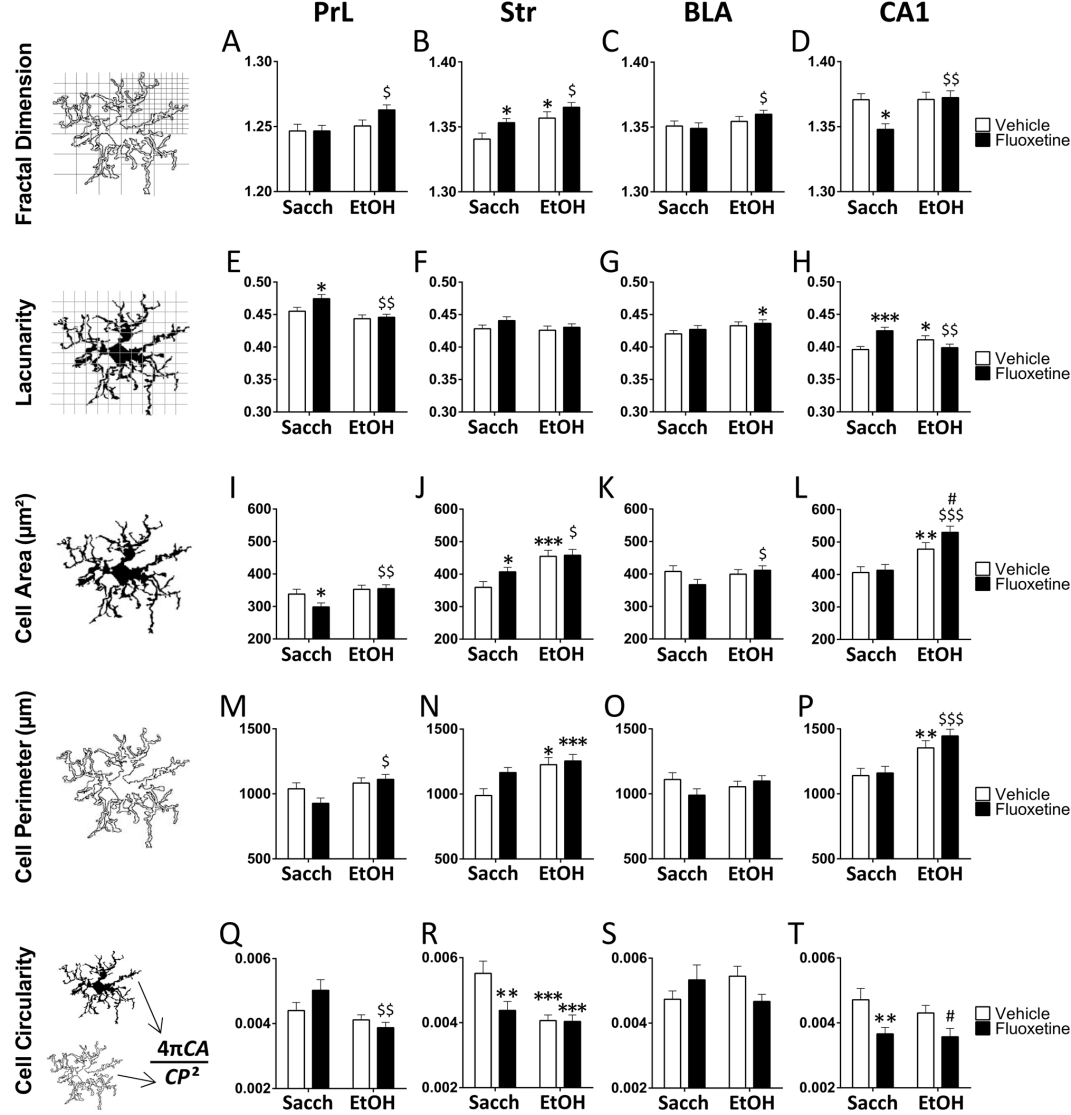
Effects of fluoxetine treatment cessation and ethanol drinking reinstatement on the morphometric parameters *fractal dimension* (**A–D**), *lacunarity* (**E–H**), *cell area* (**I–L**), *cell perimeter* (**M–P**), and *cell circularity* (**Q–T**) in the microglia of the prelimbic cortex (PrL), striatum (Str), basolateral amygdala (BLA) and hippocampal CA1. The histograms represent the mean + SEM (*n* = 50 cells per experimental group). Tukey’s test: */**/***p < 0.05/0.01/0.001 *vs*. saccharine-vehicle group; ^$/$$/$$$^*p* < 0.05/0.01/0.001 *vs*. saccharine-fluoxetine group; ^#^*p* < 0.05 *vs*. ethanol-vehicle group

Following two-way ANOVA of main factors, Tukey’s post hoc for multiple comparisons were conducted when appropriate. Ethanol increased fractal dimension (spatial comlexity) in the Str of vehicle-treated rats (*p < 0.05, Fig. 4B), as well as fractal dimension in the PrL, Str, BLA and CA1 of rats treated with fluoxetine (^$/$$^*p* < 0.05/0.01, Fig. 4A–D). Fractal dimension is increased in the Str and decreased in the CA1 of saccharine-exposed rats that were previously treated with fluoxetine (*p < 0.05, Fig. 4B, D). Ethanol decreased lacunarity (heterogeneity) in the CA1 of vehicle-treated rats (*p < 0.05, Fig. 4H), as well as lacunarity in the PrL and CA1 of fluoxetine-treated rats (^$$^*p* < 0.01, Fig. 4E, H). Fluoxetine increased lacunarity in the PrL and CA1 of saccharine-exposed rats (*/***p < 0.05/0.001, Fig. 4E, H). Microglial cell area (process branching and/or soma enlargement) was increased by ethanol in the Str and CA1 of vehicle-treated rats (**/***p < 0.01/0.001, Fig. 4J, L), as well as in the PrL, Str, BLA and CA1 of fluoxetine-treated rats (^$/$$/$$$^*p* < 0.05/0.01/0.001, Fig. 4I–L). Microglial cell area was decreased in the PrL and increased in the Str of saccharine-exposed rats that were previously treated with fluoxetine (*p < 0.05, Fig. 4I, J). Interestingly, fluoxetine increased microglial cell area in the CA1 of ethanol-exposed rats (^#^*p* < 0.05, Fig. 4L). Ethanol increased microglial cell perimeter in the Str and CA1 of vehicle-treated rats (*^/^**p < 0.05/0.01, Fig. 4N, P), as well as cell perimeter in the PrL and CA1 of fluoxetine-treated rats (^$/$$$^*p* < 0.05/0.001, Fig. 4M, P). Ethanol decreased microglial cell circularity in the Str of vehicle-treated rats (***p < 0.001, Fig. 4R), as well as cell circularity in the PrL of fluoxetine-treated rats (^$$^*p* < 0.01, Fig. 4Q). Fluoxetine also decreased cell circularity in the Str and CA1 of saccharine-exposed rats (**p < 0.01, Fig. 4R, T). Interestingly, fluoxetine specifically decreased cell circularity in the CA1 of ethanol-exposed rats (^#^*p* < 0.05, Fig. 4T).

#### Simple effect analysis of density, roughness, and convex hull area, perimeter and circularity

**Fig. 5:**
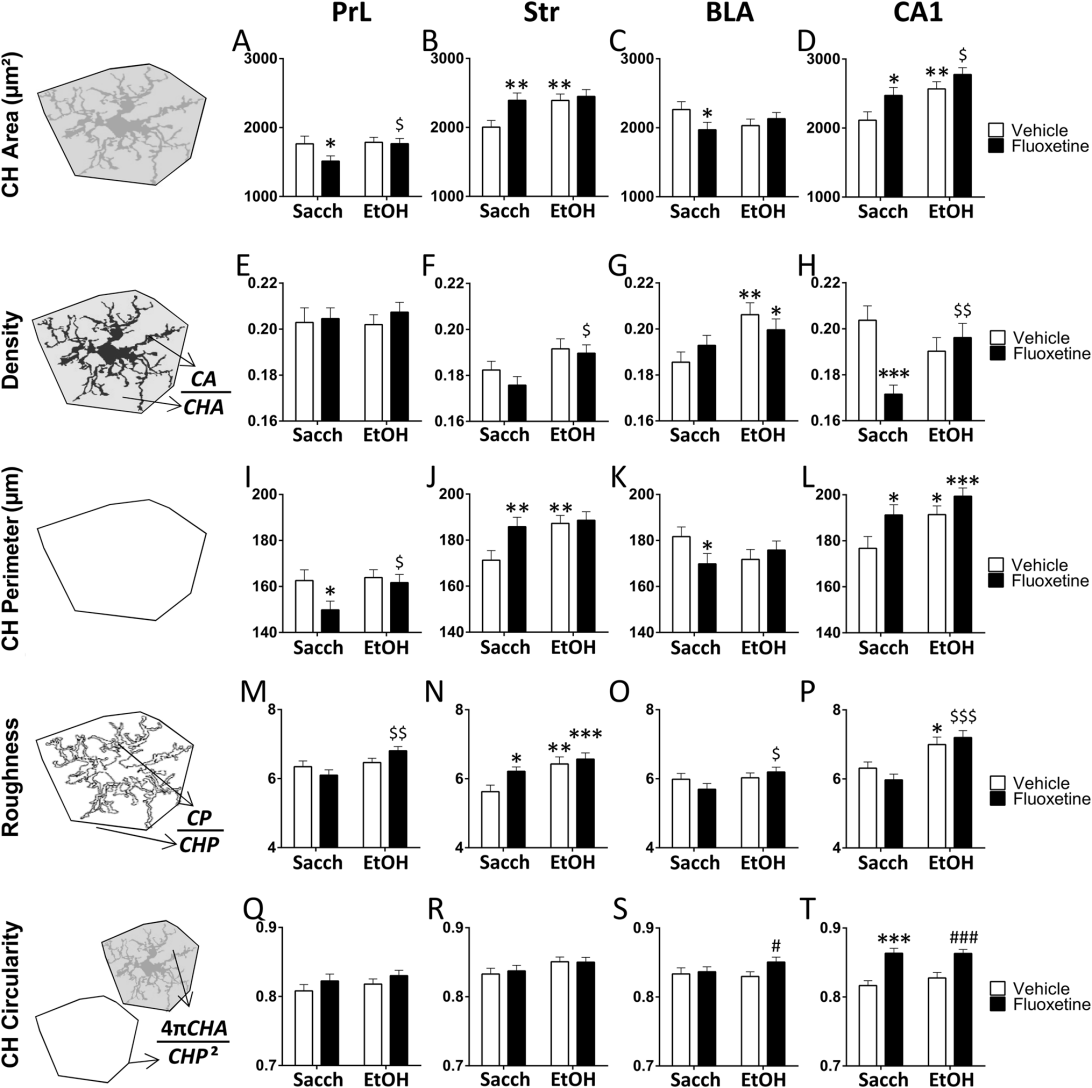
Effects of fluoxetine treatment cessation and ethanol drinking reinstatement on the morphometric parameters *CH area* (**A–D**), *density* (**E–H**), *CH perimeter* (**I–L**), *roughness* (**M–P**), and *CH circularity* (**Q–T**) in the microglia of the prelimbic cortex (PrL), striatum (Str), basolateral amygdala (BLA), and hippocampal CA1. The histograms represent the mean + SEM (*n* = 50 cells per experimental group). Tukey’s test: */**/***p < 0.05/0.01/0.001 *vs*. saccharine-vehicle group; ^$/$$/$$$^*p* < 0.05/0.01/0.001 *vs*. saccharine-fluoxetine group; ^#/###^*p* < 0.05/0.001 *vs*. ethanol-vehicle group

Following two-way ANOVA of main factors, Tukey’s post hoc for multiple comparisons were conducted when appropriate. Ethanol increased convex hull (CH) area in the Str and CA1 of vehicle-treated rats (**p < 0.01, Fig. 5B, D), as well as CH area in the PrL and CA1 of fluoxetine-treated rats (^$^*p* < 0.05, Figs. 4A and 5D). Fluoxetine decreased CH area in the PrL and BLA, but increased CH area in the Str and CA1 of saccharine-exposed rats (*^/^**p < 0.05/0.01, Fig. 5A–D). Ethanol increased density in the BLA of vehicle-treated rats (**p < 0.01, Fig. 5G), as well as density in the Str and CA1 of fluoxetine-treated rats (^$/$$^*p* < 0.05/0.01, Fig. 5F, H). In contrast, fluoxetine specifically decreased density in the CA1 of saccharine-exposed rats (***p < 0.001, Fig. 5H). Ethanol increased CH perimeter in the Str and CA1 of vehicle-treated rats (*^/^**p < 0.05/0.01, Fig. 5J, L), as well as CH perimeter in the PrL of fluoxetine-treated rats (^$^*p* < 0.05, Fig. 5I). Similar to CH area, fluoxetine decreased CH perimeter in the PrL and BLA, but increased CH perimeter in the Str and CA1 of saccharine-exposed rats (*^/^**p < 0.05/0.01, Fig. 5I–L). Ethanol increased roughness in the Str and CA1 of vehicle-treated rats (*/**p < 0.05/0.01, Fig. 5N, P), as well as roughness in the PrL, BLA and CA1 of fluoxetine-treated rats (^$/$$/$$$^*p* < 0.05/0.01/0.001, Fig. 5M, O, P). Fluoxetine specifically increased roughness in the Str of saccharine-exposed rats (*p < 0.05, Fig. 5N). Fluoxetine also increased cell circularity in the BLA and CA1 of ethanol-treated rats (^#/###^*p* < 0.05/0.001, Fig. 5S, T), as well as cell circularity in the CA1 of the saccharine-treated rats (***p < 0.001, Fig. 5T).

#### Simple effect analysis of convex hull span ratio, bounding circle diameter, maximum span across the convex hull, the ratio maximum/minimum convex hull radii and the mean radius

**Fig. 6:**
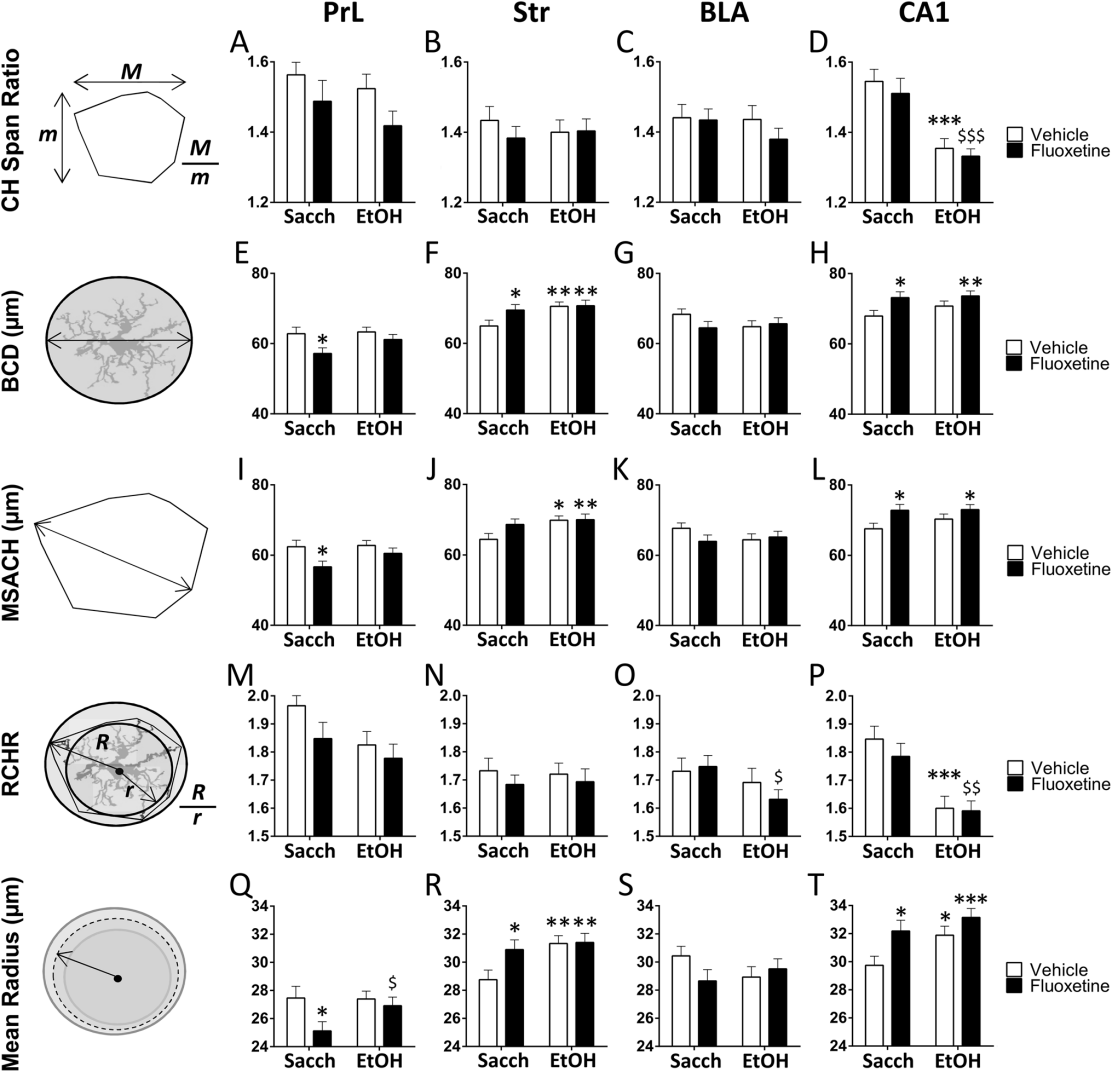
Effects of fluoxetine treatment cessation and ethanol drinking reinstatement on the morphometric parameters *CH span ratio* (**A–D**), *BCD* (**E–H**), *MSACH* (**I–L**), *RCHR* (**M–P**), and *the mean radius* (**Q–T**) in the microglia of the prelimbic cortex (PrL), striatum (Str), basolateral amygdala (BLA) and hippocampal CA1. The histograms represent the mean + SEM (*n* = 50 cells per experimental group). Tukey’s test: */**/***p < 0.05/0.01/0.001 *vs*. saccharine-vehicle group; ^$/$$/$$$^*p* < 0.05/0.01/0.001 *vs*. saccharine-fluoxetine group

Following two-way ANOVA of main factors, Tukey’s post hoc for multiple comparisons were conducted when appropriate. Ethanol specifically decreased CH span ratio in the CA1 of both vehicle- and fluoxetine-treated rats (***^/$$$^*p* < 0.001, Fig. 6D). Ethanol increased bounding circle diameter (BCD) and maximum span across the convex hull (MSACH) in the Str of vehicle-treated rats (*/**p < 0.05/0.01, Fig. 6F, J). In contrast, fluoxetine decreased BCD and MSACH in the PrL, but increased these parameters in the Str and CA1 of saccharine-exposed rats (*p < 0.05, Fig. 6E, F, H, I, L). Similar to CH span ratio, ethanol decreased the ratio maximum/minimum CH radii (RCHR) in the CA1 of both vehicle- and fluoxetine-treated rats (***^/$$^*p* < 0.001/0.01, Fig. 6P), as well as RCHR in the BLA of fluoxetine-treated rats (^$^*p* < 0.05, Fig. 6O). Ethanol increased the mean radius (MR) in the Str and CA1 of vehicle-treated rats (*/***p < 0.05/0.001, Fig. 6R, T), as well as MR in the PrL of fluoxetine-treated rats (^$^*p* < 0.05, Fig. 6Q). In contrast, fluoxetine decreased MR in the PrL, but increased MR in the Str and CA1 of saccharine-exposed rats (*p < 0.05, Fig. 6Q, R, T).

### Microglia morphology correlates with fluoxetine-induced changes in inflammatory factors and TLR4 in a brain region-specific manner

Since alcohol induced specific activation of microglia, as measured by IBA1 immunoreactivity, we evaluated whether fluoxetine treatment cessation modifies mRNA expression of inflammatory factors (cytokines and chemokines) and *TLR4* in the PrL, Str, BLA and the hippocampal CA1 region of those rats with alcohol drinking reinstatement. We also assessed whether specific morphometric features of microglia are tightly associated with changes in inflammatory factors induced by fluoxetine.

**Fig. 7:**
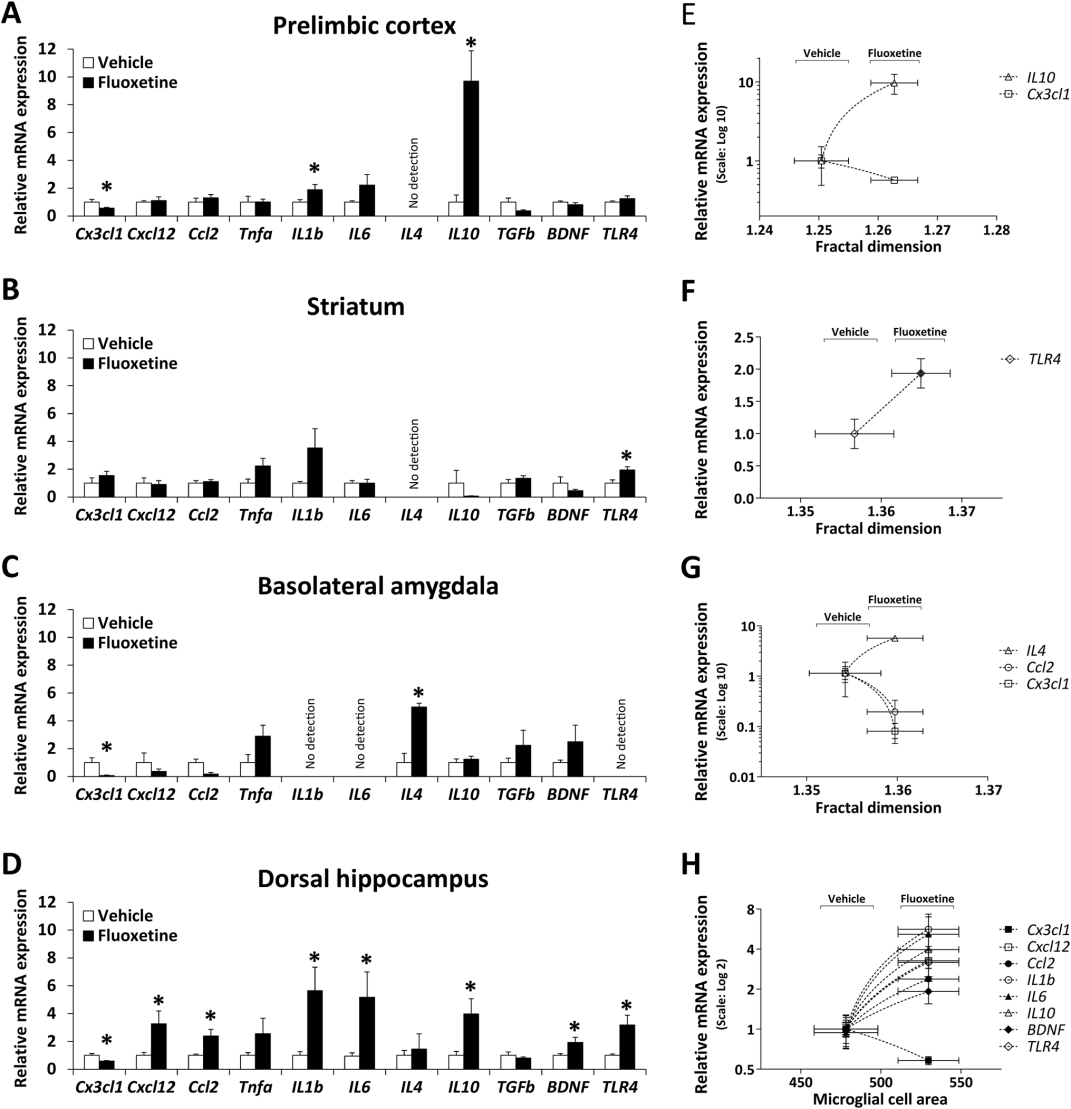
Effects of fluoxetine treatment cessation in rats with ethanol drinking reinstatement on the mRNA expression of inflammatory cytokines (*Tnfα*, *IL1β*, *IL6*, *IL4*, *IL10*, *TGFβ*, *BDNF*), chemokines (*Cx3cl1*, *Cxcl12*, *Ccl2*) and *TLR4* in the prelimbic cortex (**A**), striatum (**B**), basolateral amygdala (**C**) and dorsal hippocampus (**D**). The histograms represent the mean + SEM (*n* = 7 rats per experimental group). Student’s *t* test: *p < 0.05 *vs*. vehicle group. Correlation analysis between inflammatory factors and morphometric parameters of microglia in the prelimbic cortex (**E**), striatum (**F**), basolateral amygdala (**G**) and dorsal hippocampus (**H**) when vehicle-treated rats and fluoxetine-treated rats were faced. The scatter (XY) plots represent the means ± SEM. Plotted lines between mean points indicate that fluoxetine induces significant correlative changes between the two variables represented

Fluoxetine reduced mRNA expression of the pro-inflammatory chemokine *Cx3cl1* (*fractalkine*) and increased mRNA expression of the inflammatory cytokines *IL1b* and *IL10* in the PrL of ethanol-exposed rats (*p < 0.05, Fig. 7A). Fluoxetine only increased mRNA expression of *TLR4* in the Str of ethanol-exposed rats (*p < 0.05, Fig. 7B). Fluoxetine also reduced mRNA expression of the chemokine *Cx3cl1* and increased mRNA expression of the anti-inflammatory cytokines *IL4* in the BLA of ethanol-exposed rats (*p < 0.05, Fig. 7C). Fluoxetine also reduced mRNA expression of the chemokine *Cx3cl1* and increased mRNA expression of most of the remaining chemokines (*Cxcl12*, *Ccl2*) and cytokines (*IL1β*, *IL6*, *IL10*) analyzed, as well as *BDNF* and *TLR4*, in the dorsal hippocampus of ethanol-exposed rats (*p < 0.05, Fig. 7D).

When we analyzed whether morphometric parameters of microglia correlated with these changes of inflammatory factors induced by fluoxetine, we specifically detected that fractal dimension (microglial spatial complexity) positively correlated with mRNA expression of *IL10* (*R* = 0.67, *p* < 0.009) and negatively correlated with mRNA expression of *Cx3cl1* (*R* = − 0.53, *p* < 0.05) in the PrL (Fig. 7E). We also found that fluoxetine-induced increase in *TLR4* expression specifically correlated with higher values of fractal dimension in the Str (*R* = 0.64, *p* < 0.05, Fig. 7F). In the BLA (Fig. 7G), fractal dimension correlated with higher mRNA expression of *IL4* (*R* = 0.85, *p* = 0.0001), and lower mRNA expression of *Cx3cl1* (*R* = -− 0.60, *p* < 0.03) and *Ccl2* (R = − 0.65, *p* < 0.02). Finally, in the dorsal hippocampus (Fig. 7H), microglial cell area (process branching and/or soma enlargement) positively correlated with fluoxetine-induced increases in mRNA expression of most factors analyzed (*Cxcl2*, *Ccl2*, *IL1β*, *IL6*, *IL10*, *BDNF*, *TLR4*: *R* > 0.55, *p* < 0.04) and negatively correlated with fluoxetine-induced decreases in mRNA expression of *Cx3cl1* (*R* = − 0.66, *p* < 0.01).

## Discussion

Alcohol use disorder (AUD) results in neuroimmune consequences that contribute to depression (Neupane 2016; Erickson et al. 2019). One of the key elements of this pro-inflammatory actions of alcohol is the direct activation of TLR4 receptors in the brain (Alfonso-Loeches et al. 2010). Although the use of SSRI-antidepressants in the context of AUD when depression is present is controversial (Torrens et al. 2005), its putative anti-inflammatory pharmacotherapeutic properties have promoted its use in AUD, despite of the incongruences described on its anti-inflammatory action (Alboni et al. 2016; Chen et al. 2018).

**Fig. 8:**
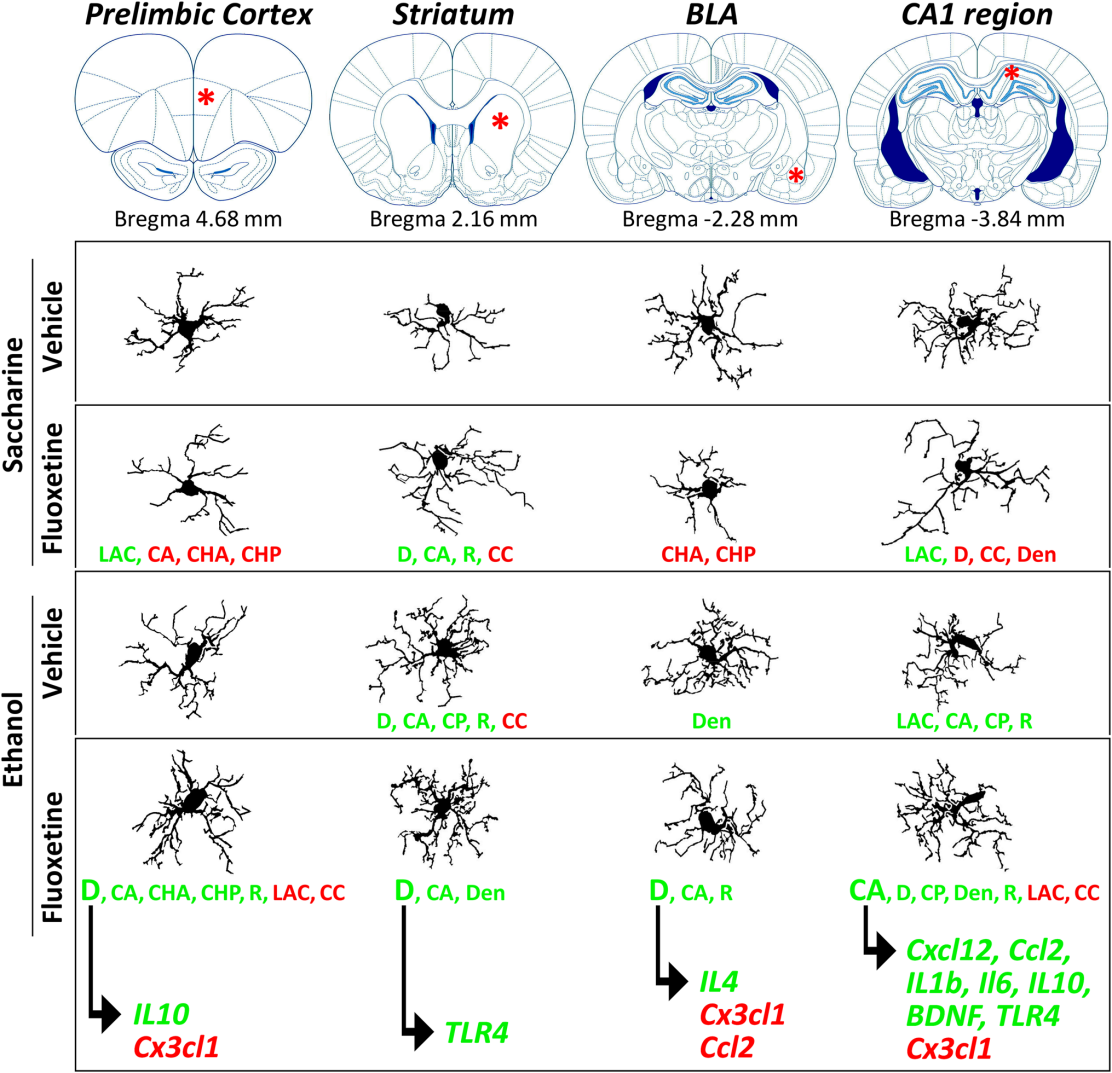
Schematic representation that summarize the main effects of fluoxetine treatment cessation and ethanol drinking reinstatement on microglial morphology and its association with reactive phenotype (inflammatory response) in each brain region analyzed

In the present study, we evaluated the inflammatory effects of alcohol drinking reinstatement (3 weeks) on microglial morphology and reactive phenotype after an experimental condition characterized by alcohol intake escalation derived from the abrupt cessation of fluoxetine treatment (10 mg/kg/day) given along a period of alcohol self-administration deprivation (14 days). Animals self-administering saccharine, receiving the same pattern of fluoxetine treatment, were used as control group. We observed that alcohol drinking reinstatement increases brain immunoreactivity for IBA-1 in all brain regions studied. IBA-1 is a protein that participates in the cytoskeleton of microglia (Ohsawa et al. 2004) and it is induced by inflammatory events, serving as an immunohistochemical marker of reactive microglia. Increases in IBA-1 immunohistochemical expression induced by ethanol are associated with changes in microglial morphology that likely depicts an activated state. Interestingly, differences in main morphometric parameters of microglia (fractal dimension, lacunarity, density, roughness, and cell area, perimeter and circularity) were observed in a brain region-specific manner (Fig. 8). Particularly, among the brain regions analyzed, the striatum and hippocampal CA1 area showed prominent alterations in microglial morphology (higher spatial complexity, branching and perimeter surface), supporting a ramified state of the microglia induced by alcohol drinking reinstatement. Despite the lack of effect of fluoxetine treatment cessation on IBA-1 immunoreactivity (a lack of effects also observed in saccharine-drinking animals), subtle changes in highly sensitive parameters of microglial morphology (fractal dimension, lacunarity, cell area and circularity, density, roughness) were detected after fluoxetine treatment cessation, resulting in a hyper-ramified, reactive-like microglia in the striatum and hippocampal CA1 area. Specifically, CA1 microglia showed an increase in cell area (process branching and/or soma enlargement) and a decrease in cell circularity (proportional index between cell area and perimeter). These morphological changes might be linked to the escalation of alcohol intake found after fluoxetine cessation (Alén et al. 2013; Suárez et al. 2020), since synapse remodeling underlying enhanced alcohol seeking/consumption behaviors needs activated microglia to readjust motivational and cognitive circuits regulating alcohol intake. This attractive hypothesis needs to be confirmed with further research.

Studies investigating the effects of alcohol exposure on microglial activation in different animal models (chronic, intermittent and binge consumption over several days, weeks and months in adult and neonatal rodents) describe increases in gene or protein expression of the microglial marker IBA-1 in the hippocampus and cerebral cortex (Ehrlich et al. 2012; Saito et al. 2010; Qin and Crews 2012a,b; Marshall et al. 2013). In agreement with these results, we also observed increases in the immunohistochemical expression of IBA-1 in PrL (also known as medial prefrontal cortex), striatum, basolateral amygdala and dorsal hippocampus of rats that were re-exposed to alcohol after a period of abstinence. Previous studies using similar animal models also demonstrated that changes in microglial morphology in brain (hippocampus, cortex, corpus callosum, cerebellum) associated with alcohol exposure consist of short, thick, few processes and large, irregular soma (Saito et al. 2010; Qin and Crews 2012a; Ward et al. 2009; Kane et al. 2011; McClain et al. 2011; Zhao et al. 2013). In the healthy brain, activated nature of resting microglia consists of multiple thin, branched processes with dynamic movements towards synapses, checking the extracellular environment for possible threats (Tremblay et al. 2011). However, a hyper-ramified state of microglia occurs in a context of pathological signals (cytokines and free radicals) induced by pathogens, neuronal damage or inflammation and consists of secondary branching and rapid, stereotyped changes in process length and reorientation towards injury (Beynon and Walker 2012). Fully activated state of microglia is characterized by a shortening and thickening in branched processes and an enlargement of the soma in an amoeboid appearance (Kreutzberg 1996). In the present study, we performed an in-depth quantitative analysis of morphometric parameters that discriminate successive changes in microglia morphology linked to different states of activation including spatial complexity (fractal dimension), heterogeneity (lacunarity), cell area, perimeter and circularity, and thickening/shortening (density and roughness) in the ramification patterns of the microglial processes. Particularly, fractal dimension seems a highly sensitive index for detecting subtle, dynamic changes in response to a diffuse injury (Soltys et al. 2001). Our analysis of microglial morphology indicates that alcohol drinking reinstatement increases microglial process complexity and thickening, specifically present in the striatum and hippocampal CA1, and reduces microglial heterogeneity (smaller gaps in space between branches), specifically present in the prelimbic cortex and hippocampal CA1, suggesting a state of activated microglia.

Microglial cells are important in generating and maintaining neuroinflammatory responses (Beynon and Walker 2012). Activated microglia are associated with the increased expression of innate immune toll-like receptors and the specific secretion of inflammatory factors (Suzumura 2009), and these signals are capable of promoting proliferation and migration of neuroimmune cells in order to allow a brain region-specific immune response (Badoer 2010). Pro-inflammatory cytokines including the interleukins IL1β and IL6, tumor necrosis factor alpha (TNFα), and transforming growth factors beta (TGFβ), as well as anti-inflammatory factors including the interleukins IL4, IL10 and the brain-derived neurotrophic factor (BDNF), ideally modulate the homeostatic inflammatory response to damage by removing pathogens or apoptotic cells (Marshall et al. 2013). However, long-term activation of microglia secreting inflammatory cytokines (or the imbalance between pro-inflammatory and anti-inflammatory cytokines) may contribute to the pathologic condition of AUD (Crews et al. 2011).

Among the neuropsychiatric disorders involving behavioral and cognitive dysfunction, depression is the most prevalent comorbidity in AUD patients. Similar to alcohol actions, over-activation of the neuroimmune system and microglial mechanisms that contribute to inflammation play a critical role in major depressive disorders (Rahman and Alzarea 2019). Expression of the microglial marker IBA-1 and inflammatory cytokines (IL1β, TNFα, BDNF) are up-regulated in the brain of patients with major depression (Rahman and Alzarea 2019; Torres-Platas et al. 2014) and in animal models of depressive-like behavior (Kreisel et al. 2014; Tomaz et al. 2020). Irrespective of whether inflammation promotes susceptibility to depression, controlling inflammation by antidepressants may provide a comprehensive therapeutic benefit (Beurel et al. 2020). However, conventional SSRI antidepressants fail to respond to immunotherapies involving depressive disorder, demonstrating no, anti- or even pro-inflammatory effects (Hannestad et al. 2011; Kim et al. 2013; Chen et al. 2018; Tomaz et al. 2020; Yoshimura et al. 2013). Experimental studies reflecting the incongruence of clinical outcomes hypothesize that an interplay between SSRIs and the quality of the environmental conditions (Alboni et al. 2016,2017) may support the divergent effects of SSRI treatment on the inflammatory response (Tynan et al. 2012; Horowitz et al. 2014; Lu et al. 2017; Zhang et al. 2019). Regarding our findings on microglial morphology, fluoxetine treatment cessation induced subtle changes in main morphometric parameters that indicate higher spatial complexity (fractal dimension) and lower heterogeneity (lacunarity), as well as increases in process thickening and shortening (density and roughness), effects that are also more prominent in the striatum and hippocampal CA1. Together, these morphological changes suggest an increase in process branching and complexity that depicts a hyper-ramified, reactive state of microglia associated with fluoxetine treatment cessation, suggesting a microglial behavior that can be functionally over-reactive to that induced by alcohol.

Besides microglial morphology, our investigation also evidences that alcohol drinking reinstatement may contribute to support an imbalanced inflammatory response of SSRI antidepressants after the abrupt cessation of treatment during alcohol abstinence. Results indicate that fluoxetine treatment cessation in rats with alcohol drinking reinstatement is associated with changes in the expressions of both pro-inflammatory and anti-inflammatory cytokines (*TNFα*, *IL1β*, *IL*6, *IL4*, *IL10*, *TGFβ*, *BDNF*), most of the chemokines (*Cxcl12*, *Ccl2, Cx3cl1*) and *TLR4* in a brain region-specific manner, having the dorsal hippocampus a more significant neuroimmune response. It is of particular interest the lower mRNA expression of the pro-inflammatory chemokine *Cx3cl1* (*fractalkine*) in the PrL, BLA and dorsal hippocampus, as well as the higher mRNA expression of *TLR4* in the Str and dorsal hippocampus of those ethanol-exposed rats whose fluoxetine treatment was abruptly ceased. We next wonder whether fluoxetine-induced changes in inflammatory factors and *TLR4* are tightly related to microglial morphology. Overall, we found that an altered mRNA expression, induced by fluoxetine, in most of the inflammatory factors correlates with increases in fractal dimension (*D*), a morphological index of microglial spatial complexity (Fig. 8). Fractal dimension is a highly sensitive parameter that mirrors secondary branching and process readjusting usually observed during the hyper-ramified state of the activated microglia (Beynon and Walker 2012). Interestingly, we highlight that fluoxetine-induced decreases in *Cx3cl1* correlate with fractal dimension (spatial complexity) in the PrL and BLA, and microglial cell area (process branching) in the dorsal hippocampus of ethanol-exposed rats. The inverse association between *Cx3cl1* expression and spatial complexity and cell area of microglia agrees with the recently known role of CX3CL1 signaling on microglia-mediated synapse remodeling (Gunner et al. 2019) and the subsequent declines in cognitive behavior in mice lacking *Cx3cl1* (Winter et al. 2020). Moreover, microglial cell area correlates with fluoxetine-induced increases in the remaining chemokines (*Cxcl2*, *Ccl2*) and most of the inflammatory cytokines (*IL1β*, *IL6*, *IL10*) in the dorsal hippocampus. Moreover, in the dorsal hippocampus (Fig. 8), *TLR4* mRNA expression positively correlates with microglial cell area. It is of particular relevance that fluoxetine-induced increases in striatal *TLR4* specifically correlates with an increase in fractal dimension (D), a morphometric index that depicts complexity in process branching. The positive association between spatial complexity of microglia and *TLR4* expression supports previous reports addressing a role of TLR4 receptors in microglial interactions with neurons including synaptic connections induced by ethanol (Montesinos et al. 2018). Focusing on drinking behavior, these results also suggest that changes in microglial morphology and reactive phenotype, characterized by the expression of ethanol-specific TLR4 signaling of inflammatory and chemoattractant factors, may underlie fluoxetine-induced escalation of alcohol consumption as was previously described by our group (Alén et al. 2013; Suárez et al. 2020). Following this promising finding, further studies would be needed to determine whether the effect of SSRI antidepressants on microglial morphology linked to TLR4/inflammatory signaling in the dorsal hippocampus may contribute to modulate cognitive impairment induced by long-term alcohol consumption.

There are some limitations on the present study that have to be highlighted. First, the data obtained using only male rats need to be extended towards females in order to confirm both fluoxetine cessation-induced escalation in alcohol consumption and the alterations in microglial reactivity described in the present study. The fact that fluoxetine activates anti-inflammatory mechanisms demands additional controls where the maintenance of fluoxetine treatment after resuming alcohol-self administration is present. Moreover, since fluoxetine induces escalation, a control pair-feeding with alcohol should be needed to identify dose effects. Finally, the apparent lack of direct effect of TLR4 signaling on ethanol excessive drinking (Harris et al. 2017) suggests the need of further studies to evaluate alternative mechanisms where the involvement of TLR4 in local neuroimmune/inflammatory responses may regulate ethanol-induced sedation via the histaminergic system (Ma et al. 2019).

In conclusion, our study provides evidence of brain region-specific effects of the SSRI fluoxetine treatment cessation on microglia morphology and neuroimmune/inflammatory response to alcohol drinking reinstatement. Data suggest that fluoxetine might modify neuroinflammation associated with alcohol consumption. They also remark the need to avoid abrupt cessation of fluoxetine that might produce relevant changes on neuroinflammatory mechanisms activated by alcohol intake, a fact that might be linked to the escalation of alcohol consumption and might also results in additional comorbidities. Results shed light on the complexity in understanding of the SSRI antidepressant actions on alcohol relapse and may help to develop personalized strategies using SSRI antidepressants associated with anti-inflammatory medications as new therapeutic strategies for AUD and depression. Clinical studies using AUD patients with co-occurring depressive symptoms are needed to address the efficacy of these treatments.

## Data Availability

The data that support the findings of this study are available on reasonable request from the corresponding author.

## Abbreviations

*BCD*: Bounding circle diameter
*BDNF*: Brain-derived neurotrophic factor
*BLA*: Basolateral amygdala
*CA*: Cell area
*CA1/CA3*: *cornu ammonis* Subfields 1 and 3 of the hippocampus
*CC*: Cell circularity
*CCL2/MCP1*: Monocyte chemoattractant protein-1
*CH*: Convex hull area (CHA), circularity (CHC), perimeter (CHP), and span ratio (CHSR)
*CP*: Cell perimeter
*CX3CL1*: Fractalkine
*CXCL12/SDF1*: Stromal cell derived factor 1
*D*: Fractal dimension
*DG*: Dentate gyrus of the hippocampus
*Gapdh*: Glyceraldehyde-3-phosphate dehydrogenase
*Gcl*: Granular cell layer
*IBA-1*: Allograph inflammaroty factor 1 or ionized calcium-binding adapter molecule 1
*IL1β*: Interleukin 1 beta
*IL4*: Interleukin 4
*IL6*: Interleukin 6
*IL10*: Interleukin 10
*Lac*: Lacunarity (Ʌ)
*MR*: The mean radius
*MSACH*: Maximum span across the convex hull
*Pcl*: Polymorphic cell layer or hilus
*PrL*: Prelimbic cortex (also known as medial prefrontal cortex)
*RCHR*: The ratio maximum/minimum convex hull ratii
*SR*: Stratum radiatum
*Str*: StriatumTGFB1: transforming growth factor beta 1
*TLR4*: Toll-like receptor 4
*TNFα*: Tumor necrosis factor
